# P2X7 on Mouse T Cells: One Channel, Many Functions

**DOI:** 10.3389/fimmu.2015.00204

**Published:** 2015-05-19

**Authors:** Björn Rissiek, Friedrich Haag, Olivier Boyer, Friedrich Koch-Nolte, Sahil Adriouch

**Affiliations:** ^1^Insitute of Immunology, University Medical Center, Hamburg, Germany; ^2^Department of Neurology, University Medical Center, Hamburg, Germany; ^3^U905, INSERM, Rouen, France; ^4^Institute for Research and Innovation in Biomedicine (IRIB), Normandy University, Rouen, France; ^5^Department of Immunology, Rouen University Hospital, Rouen, France

**Keywords:** P2X7, P2RX7, ATP, T cells, purinergic signaling

## Abstract

The P2X7 receptor is an adenosine triphosphate (ATP)-gated cation channel that is expressed by several cells of the immune system. P2X7 is best known for its proinflammatory role in promoting inflammasome formation and release of mature interleukin (IL)-1β by innate immune cells. Mounting evidence indicates that P2X7 is also an important regulatory receptor of murine and human T cell functions. Murine T cells express a sensitive splice variant of P2X7 that can be activated either by non-covalent binding of ATP or, in the presence of nicotinamide adenine dinucleotide, by its covalent ADP-ribosylation catalyzed by the ecto-ADP-ribosyltransferase ARTC2.2. Prolonged activation of P2X7 by either one of these pathways triggers the induction of T cell death. Conversely, lower concentrations of ATP can activate P2X7 to enhance T cell proliferation and production of IL-2. In this review, we will highlight the molecular and cellular consequences of P2X7 activation on mouse T cells and its versatile role in T cell homeostasis and activation. Further, we will discuss important differences in the function of P2X7 on human and murine T cells.

## Mechanisms Leading to Activation of P2X7 by Extracellular ATP or NAD^+^ on Mouse T Cells

**Figure 1:**
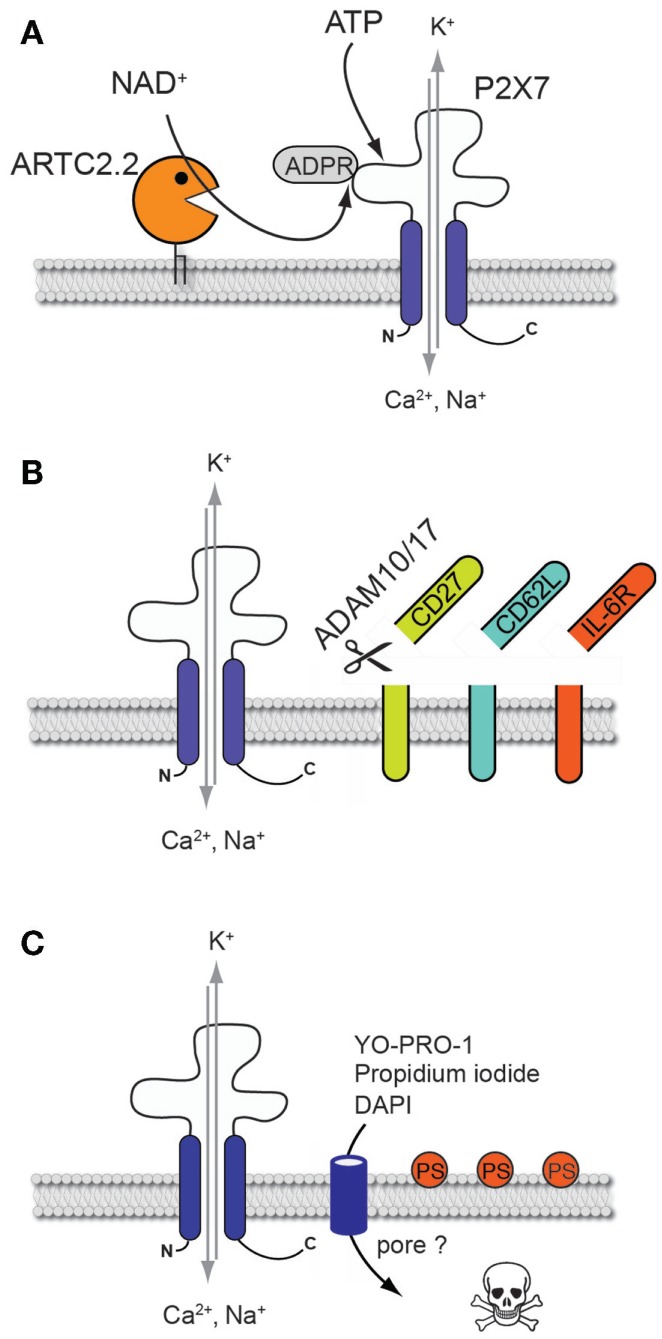
**Cellular consequences of P2X7 activation on mouse T cells**. **(A)** Non-covalent binding of ATP or covalent ARTC2.2-mediated ADP-ribosylation results in P2X7 channel gating and in the influx of calcium and sodium ions and the efflux of potassium. **(B)** P2X7 activation triggers the activation of ADAM10 and ADAM17 that catalyze the shedding of the ectodomains of various cell surface proteins such as CD27, CD62L and IL-6R. **(C)** P2X7 activation leads to phosphatidylserine (PS) externalization, formation of a non-selective membrane pore allowing the uptake of fluorescent molecules such as DNA staining dyes, and ultimately to cell death.

The family of ionotropic P2X receptors comprises seven members that are able to form trimeric ion channels reactive to extracellular adenosine triphosphate (ATP). In the context of the immune system, P2X7 is best known for its role in promoting inflammasome formation and release of the proinflammatory interleukin 1β (IL-1β) from innate immune cells such as macrophages and monocytes after exposure to lipopolysaccharide and ATP (1). Further, P2X7 has also been identified as an important regulator of mouse T cell functions. P2X7 triggered by extracellular ATP induces the formation of a non-selective cation channel, resulting in the influx of calcium and sodium ions as well as the efflux of potassium. Interestingly, P2X7 can also be triggered via an ATP-independent pathway that has been discovered and characterized on mouse T cells. The mechanism involves the ecto-ADP-ribosyltransferase ARTC2.2 which, in the presence of its substrate nicotinamide adenine dinucleotide (NAD^+^), catalyzes the ARTC2.2-dependent ADP-ribosylation of P2X7 at an arginine residue at position 125 at the edge of the ATP-binding pocket in the extracellular domain of the protein (Figure 1A) (2, 3). This covalent posttranslational modification occurs even at 4°C, however gating of P2X7 is triggered only at 37°C. Since NAD^+^ is released during cell preparation, alterations of T cell phenotype and function due to NAD^+^-dependent ADP-ribosylation of P2X7 can easily be evidenced on freshly harvested T cells extracted from lymphoid organs following their re-incubation at 37°C (4–6). Importantly, NAD^+^-dependent activation of P2X7 can only be observed with mouse T cells but not with human T cells that lack ADP-ribosyltransferase activity due to premature stop codons in the orthologous pseudogene (7).

Interestingly, NAD^+^-dependent activation of P2X7 by ADP-ribosylation provides a long-lasting activation signal, even after a brief exposition to relatively low concentrations of extracellular NAD^+^ (i.e., 1–30 μM) (8). In contrast, P2X7 activation by the non-covalent binding of ATP necessitates its continuous presence in the extracellular milieu at sufficient concentrations (i.e., above 100 μM) (4). It is therefore suspected that regulation of T cell phenotype, function, survival, and differentiation by either of these pathways occurs in different pathophysiological situations that are not necessarily redundant. Both activation pathways are tightly regulated by ectoenzymes, notably by the ATP-degrading ectonucleoside triphosphate diphosphohydrolase CD39 and the NAD^+^-degrading ecto-NAD^+^-glycohydrolase CD38, respectively (9–11). Of note, these enzymes have been demonstrated to participate in the regulation of immune responses and T cell activation and function. For instance, CD39 has been implicated in the suppressive functions of regulatory T cells (Tregs) and CD38 in the activation of human T cells via the generation of cyclic ADP-ribose (cADPR) that can cause the release of Ca^2+^ from intracellular ryanodine-sensitive stores (12, 13). Additionally, the activity of these enzymes participates in the maintenance of low extracellular ATP or NAD^+^ concentrations in biological fluids. Under steady-state conditions, their concentrations in the serum have been estimated in the submicromolar range (14, 15). Therefore, activation of P2X7 by ATP or NAD^+^ is limited to situations where intracellular pools of nucleotides are released in substantial amounts. Interestingly, ATP has been demonstrated to be released in the tumor microenvironment in concentrations that are fully compatible with the *in vivo* direct activation of P2X7 at the surface of innate immune cells and tumor-infiltrating T cells (16, 17). Whether NAD^+^ can also be released in this situation is presently not known, but NAD^+^ has been demonstrated to be released within inflammatory sites and to affect the phenotype and survival of T cells located in the proximal draining lymph nodes via the ARTC2.2/P2X7 pathway (18).

## Molecular and Cellular Consequences of P2X7 Activation on Mouse T Cells

Activation of P2X7 on T cells by extracellular ATP or following NAD^+^-dependent ADP-ribosylation induces changes in cell volume and composition of the plasma membrane. T cells respond to ATP stimulation with rapid sequential shrinkage and swelling and externalization of phosphatidylserine (PS) onto the outer leaflet of the plasma membrane (Figure 1C) (19, 20). Analyses of thymocytes revealed that PS externalization is related to the influx of both, calcium and sodium ions, inhibiting aminophospholipid translocases responsible for maintaining PS at the inner leaflet of the plasma membrane, and simultaneously activating scramblases which catalyze the bidirectional transbilayer movement of PS (21–23). Externalization of PS is regarded as an early indicator of the induction of apoptosis. Prolonged activation of P2X7 indeed triggers T cell death which can be visualized by staining with DNA-binding dyes such as propidium iodide following loss of membrane integrity (4). Interestingly, PS exposure after ATP stimulation is reversible if the ATP is removed within the first 30 min of exposure (24). Conversely, PS exposure following ADP-ribosylation of P2X7 is not reversed by removing the ARTC2 substrate NAD^+^ (2).

Another hallmark of P2X7 activation is the formation of membrane pores permeable to molecules up to a molecular weight of 900 Da. Pore formation has been functionally linked to the long intracellular C-terminus region of P2X7 (25). However, whether P2X7 itself directly mediates pore formation by channel dilation or whether other P2X7-associated proteins such as pannexin 1 form the pores is still a matter of debate. Interestingly, inhibition of pannexin-1 significantly reduced ATP-induced mouse T cell death (26). This suggests that drastic elevation of intracellular Ca^2+^, either through P2X7 itself or via other associated non-selective pore-forming proteins, may represent an essential common event triggered in the early phase leading to cell death. A recently published study identified the phospholipid scramblase anoctamin 6 (ANO6), another non-selective cation channel, as a new key player in the formation of membrane pores following P2X7 activation on macrophages (27). Whether ANO6 is also involved in P2X7-mediated pore formation in T cells needs to be further investigated.

P2X7 activation is associated with a rapid change in T cell surface phenotype. The mechanism involves the activation of the membrane-associated metalloproteases ADAM10 and ADAM17 that catalyze the shedding of the ectodomains of various cell surface proteins such as CD62L (28), CD27 (29), and IL-6R (30) (Figure 1B). Hence, on the cellular level, activation of P2X7 on T cells results in the triggering of multiple signaling pathways that affect cell morphology, phenotype, and viability. In the following sections, we will discuss the impact of P2X7-mediated cell death on T cell function and homeostasis.

## Allelic and Splice Variants Affect the Functionality of P2X7

In the mouse, single nucleotide polymorphisms (SNP) and alternative splicing result in the expression of different P2X7 variants (1, 31–35). An allelic variant of P2X7 located in the long C-terminal cytosolic tail was discovered in widely used C57BL/6 laboratory mice. The 451L variant found in this strain affects the function of the receptor when expressed by HEK cells as compared to the P451 P2X7 allelic variant found in the BALB/c strain. Side-by-side comparison of T cells from both strains concordantly showed impaired functional responses of the 451L variant to P2X7 stimulation leading to the conclusion that the P451L SNP affects the functionality of P2X7 expressed by conventional mouse T cells (36). However, later analyses using anti-P2X7 antibodies suitable for flow cytometry analysis demonstrated that conventional T cells from C57BL/6 display very low cell surface expression of P2X7 which possibly accounts for their relative insensitivity to P2X7 ligands (5). The situation was further complicated by the discovery of two alternatively spliced variants termed P2X7a and P2X7k with different sensitivity to P2X7 agonists. Structurally, the P2X7k variant differs from the previously described P2X7a by 42 amino acids encompassing the N-terminal cytosolic region and a part of the first transmembrane domain (25). Functionally, the P2X7k variant was found to be 8–10 times more sensitive to P2X7 agonists than the classical P2X7a variant (37). Also, we and others have shown that P2X7k is the predominant variant expressed by T cells, whereas P2X7a is the predominant variant expressed by macrophages (38, 39). Interestingly, differential expression of these variants on immune cells explains earlier observed differences in the sensitivities of T cells and macrophages to extracellular ATP (40). Additionally, expression of both variants in HEK cells revealed that only the P2X7k variant can be efficiently activated by ADP-ribosylation even though both variants are equally modified by ARTC2.2 in the presence of extracellular NAD^+^ (38, 39). This was attributed to a higher stability of the activated state of this variant compared with P2X7a. Further, these studies also demonstrated that the P451L SNP described earlier only impairs the functionality of P2X7a but not the functionality of the highly sensitive P2X7k variants. Hence, lower sensitivity of conventional T cells from C57BL/6 mice to ATP and NAD^+^ most likely reflects lower cell surface expression of P2X7k rather than impaired functionality due to the 451L SNP that would rather affect cells like macrophages and dendritic cells (DCs) that express the P2X7a splice variants.

Unexpectedly, the characterization of mouse P2X7k splice variants brought new insights to some apparently conflicting results using P2X7-deficient mice. Two distinct P2X7-deficient lines have been generated and were used to decipher the function of P2X7 *in vivo*. One P2X7-deficient mouse, developed by Pfizer, was generated by disrupting the last exon coding for the long C–terminal cytoplasmic tail (41). In the other P2X7-deficient mouse, generated by GlaxoSmithKline (GSK), the *lacZ* gene was inserted at the beginning of exon 1 (42–44). However, two independent studies revealed that the latter mice do express a functional P2X7 receptor on T cells, whereas DCs, macrophages, and neurons were effectively deficient in P2X7 functional activity (37, 45). Later studies revealed that this is due to the preferential use of the P2X7k variant in T cells which escapes exon 1 targeted disruption (i.e., using an alternative exon 1′) in the GSK knockout strain (37, 38). This finding may be important for our understanding of the role of this receptor *in vivo* and may help reinterpret some contradictory results. For instance, autoimmune encephalomyelitis was found to be exacerbated in the Pfizer knockout strain, whereas it was found to be attenuated in GSK knockout strain (46, 47). Future studies using tissue-specific P2X7 disruption in lymphoid, myeloid, or oligodendrocytes cells should help clarify this issue.

## P2X7 Expression on T Cells of Different Differentiation and Maturation Stages

P2X7 expression on mouse T cells depends on differentiation and maturation stage. Cell death can be induced in a fraction of thymocytes by extracellular ATP suggesting that *P2rx7* gene expression is induced in T cells during their differentiation in the thymus (48). The precise physiological role of P2X7 on the selection of the T cell repertoire is not known but ATP has been suggested to play a role in thymocyte death by neglect and in the thymic differentiation of γδ T cells (49). Our own early studies have revealed that single positive CD4^+^ or CD8^+^ thymocytes express higher levels of surface P2X7 than less differentiated double positive cells suggesting that P2X7 expression correlates with T cell maturation. Accordingly, peripheral T cells displayed higher sensitivity to ATP and NAD^+^ than thymocytes (2, 50). On mature peripheral T cells, CD4^+^ T cells display slightly higher surface levels of P2X7 as compared to CD8^+^ T cells that may account in part for their higher sensitivity to extracellular ATP and NAD^+^. Interestingly, P2X7 surface expression also seems to be regulated by activation. Indeed, cells expressing T cell activation markers display lower P2X7 levels than their naive counterparts (18). Also, recently activated T cells display a lower level of ARTC2.2 possibly as a result of its shedding from the cell surface by metalloproteases (51). Of note, the relative expression levels of P2X7a and P2X7k variants during cell differentiation in the thymus, in mature CD4^+^ or CD8^+^ T cell lineages, or after activation are currently not known but may possibly regulate the sensitivity to extracellular ATP and NAD^+^.

## P2X7-Mediated Cell Death Affects the Quality of *Ex Vivo* Murine T Cell Preparations

**Figure 2:**
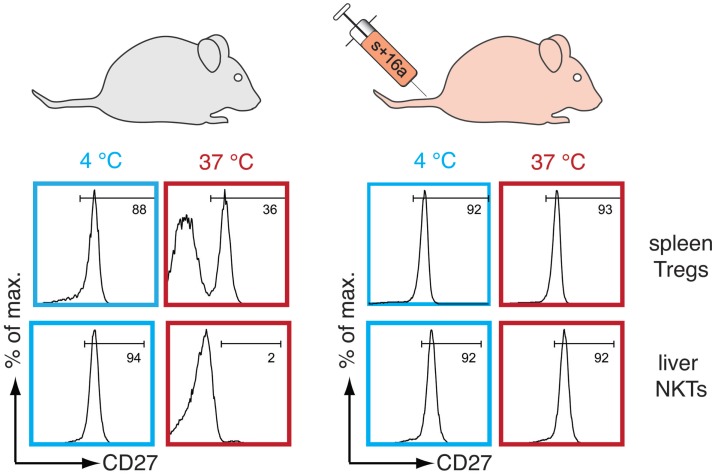
**P2X7-dependent alteration of T cells can be prevented by blocking the activity of ARTC2.2**. Freshly prepared spleen CD4^+^Foxp3^+^ Tregs and liver CD3^+^CD1d-tetramer^+^ NKT cells remain unaffected when kept at 4°C but rapidly shed CD27 when re-incubated at 37°C due to NAD^+^ release during cell preparation and ARTC2.2-mediated ADP-ribosylation of P2X7 (left panel). These cells also rapidly shed CD62L and expose phosphatidylserine at their cell surface (not illustrated here). Intravenous injection of an anti-ARTC2.2 blocking nanobody (s + 16a) before harvesting of the organs prevents ADP-ribosylation of P2X7 during cell manipulation and P2X7-dependent CD27 shedding when cells are re-incubated at 37°C (right panel).

Several studies have shown that the expression levels of P2X7 and ARTC2.2 differ among T cell subsets (52, 53). Tregs and liver natural killer T cells (NKTs) exhibit a higher cell surface expression of P2X7 as compared to conventional naïve CD4^+^ T cells, and this is associated with a higher sensitivity to ATP- and NAD^+^-mediated cell death (5, 6, 54, 55). High sensitivity to extracellular NAD^+^ negatively correlates with the survival and function of *ex vivo* freshly prepared Tregs and liver NKTs. This has a significant impact on *in vitro* assays and on adoptive transfer experiments (6). NAD^+^ is released in sufficient amounts during organ collection and cell preparation to ADP-ribosylate P2X7 on a large fraction of Tregs and on the majority of liver NKTs even when cells are prepared on ice. This leads to the induction of P2X7-mediated cell death when the cells are returned to 37°C either *in vitro* or after their adoptive transfer into living animals (Figure 2). To overcome this detrimental effect, an ARTC2.2-antagonizing nanobody (clone s + 16a) has been developed (56) and was shown to prevent ADP-ribosylation-related detrimental effects induced during *ex vivo* cell preparation when injected a few minutes before sacrificing the mice (Figure 2). This markedly preserved the viability, the phenotype and the function of harvested Tregs and NKTs and improved subsequent downstream applications *in vitro* and *in vivo* (6).

## P2X7-Mediated Cell Death Regulates Mouse T Cell Homeostasis *In vivo*

P2X7-dependent cell death plays an important role in the homeostatic regulation of diverse T cell populations during steady state and in pathophysiological contexts. The endogenous sources of extracellular ATP and NAD^+^ remain an important question when addressing the role of P2X7 *in vivo*. Obviously, ATP and NAD^+^ can be passively liberated from damaged cells in traumatic situations (4). However, extracellular nucleotides can also be liberated from living cells in various situations (e.g., during cell activation, during autophagy, in conditions of shear-force stress, oxidative stress, or from transformed cells) through hemichannels such as pannexin 1 or connexin 43 and possibly through other still unknown mechanisms (57, 58). In a proof-of-principle study, we previously showed that NAD^+^ is released at inflammatory sites and even can gain access to the local draining lymph nodes (18). This led to P2X7-dependent phenotypic changes and to the depletion of a fraction of naïve T cells that were detectable in wildtype (WT) mice and, more prominently, in CD38-deficient mice, in which NAD^+^ has a longer half-life in the extracellular compartment.

Given the higher sensitivity of Tregs and NKTs to ATP- and NAD^+^-mediated cell death, the role of P2X7 in their homeostatic maintenance *in vivo* was studied (5, 6, 54, 55). P2X7 negatively regulates the number of peripheral Tregs and was therefore suggested to participate in their homeostasis. Indeed, P2X7-deficient mice exhibit higher numbers of Tregs in the spleen compared to their WT counterparts. Conversely, CD38-deficient mice display lower numbers of Tregs in the spleen, indirectly suggesting that extracellular NAD^+^ also regulates Treg homeostasis (5). Whether the increased number of Tregs in P2X7-deficient mice is related to ATP- and/or NAD^+^-induced cell death needs to be further investigated. Interestingly, CD38 deficiency similarly affects the number of NKTs. This was observed in diabetes-prone NOD mice, in which CD38 deficiency was accompanied by an accelerated onset of diabetes (59). We could further show that *in vivo* blockade of the ARTC2.2/P2X7 pathway using an ARTC2.2-antagonistic nanobody restored normal numbers of NKT cells in these mice suggesting that extracellular NAD^+^ indeed can influence NKT cell homeostasis (59).

The high sensitivity of Tregs and NKTs to NAD^+^-induced P2X7-dependent cell death has been exploited to deplete these cells *in vivo*. Injection of NAD^+^ into mice depleted NKT cells and protected mice from concanavalin A (ConA)-mediated autoimmune hepatitis. This was linked to a transient decrease in the number of liver NKT cells, which otherwise respond to ConA with a massive production of proinflammatory cytokines resulting in severe liver damage (55, 60). Similarly, a single intravenous injection of NAD^+^ decreased the number of peripheral Tregs by 80%. When applied 24 h before tumor engraftment, this unleashed an effective anti-tumor immune response leading to tumor rejection (5).

The role of P2X7 in the tumor microenvironment has been investigated in two recent publications addressing tumor promotion and tumor growth in P2X7-deficient mice. In one of these studies, genetic and pharmacological inactivation of P2X7 was demonstrated to increase tumor development in a model of colitis-associated cancer (61). Interestingly, this was associated with alteration of the number and the quality of tumor-infiltrating immune cells and, notably, by the accumulation of Tregs within the colonic lesions. Their accumulation in the tumor of P2X7-deficient is compatible with their high sensitivity to P2X7-dependent cell death that would partially prevent their early accumulation within the tumor microenvironment of WT mice. However, only naive Tregs, but not activated Tregs that are resistant to P2X7-dependent cell death, would be affected by such a mechanism. Another mechanism could additionally rely on the more efficient priming of anti-tumor CD8^+^ T cell response in WT mice through a P2X7/NLRP3-dependent maturation of DCs and effective tumor-antigen presentation to the adaptive immune system that could also influence the effector/Tregs ratio found within the tumors (62). This is supported by the finding of the second study showing accelerated tumor progression in P2X7-deficient host mice inoculated with B16 melanoma or CT26 colon carcinoma cell lines (63). In this study, DCs from P2X7-deficient host mice were found to be unresponsive to stimulation with tumor cells *in vitro* which correlates *in vivo* with a lower intratumoral level of IL-1β and a lower infiltration by immune cells and notably by CD8^+^ cytotoxic T cells. Collectively, these studies highlights the importance of P2X7 expression on Tregs and DCs in the anti-tumor immune response and in the restriction of tumor growth.

Interestingly, intestinal CD8^+^ T cells (both conventional CD8αβ^+^ as well as unconventional CD8αα^+^ T cells) express higher levels of surface P2X7 as compared to splenic CD8^+^ T cells (64). Accordingly, intestinal CD8^+^ T cells are more susceptible to ATP- and NAD^+^-induced cell death *in vitro*. Similarly, injection of NAD^+^ leads to a depletion of these cells *in vivo*. These data suggested for the first time that P2X7 expression level is not only regulated during T cell differentiation but also by tissue-derived specific factors. For intestinal CD8^+^ T cells, intestinal retinoic acid (RA) was suggested to contribute to P2X7 and ARTC2.2 upregulation (64). Indeed, DCs located in the gut-associated mesenteric lymph nodes and Peyers Patches (PPs) express retinal dehydrogenase and generate retinoic acid from orally derived vitamin A (65, 66). Accordingly, incubation of splenic CD8^+^ T cells with RA significantly upregulated the surface levels of P2X7 and ARTC2.2. A functional implication of the higher P2X7 expression on intestinal CD8^+^ cells was studied in the context of intestinal *Listeria monocytogenes* (LM) infection. P2X7-deficient mice infected with LM exhibit higher frequencies of antigen-specific IFNγ^+^ CD8^+^ T cells in the intestinal epithelium compared to infected WT mice. Whether this is related to enhanced survival of intestinal CD8^+^ T cells to ATP and/or NAD^+^ derived from the inflammatory site or to other mechanisms needs to be further investigated (64).

Importantly, recent work by the group of Fabio Grassi highlighted the role of P2X7 in regulating the survival of T follicular helper cells (Tfh) in the gut environment (67). Tfh cells in the PPs promote the secretion of high-affinity IgA, which affects intestinal homeostasis, mucosal defense, and the composition of intestinal microbiota. P2X7 is expressed at high levels on Tfh located in PPs and critically regulates their survival and/or function. Indeed, under steady-state conditions, P2X7-deficient mice displayed increased numbers of Tfh and germinal centers in their PPs along with an enhanced production of high-affinity IgA, which resulted in an alteration of the microbiota. Consequently, P2X7-deficient mice exhibited lower serum IgM concentrations and increased susceptibility to polymicrobial sepsis (67). Whether P2X7-dependent control of Tfh homeostasis in PPs is regulated by local release of ATP and/or NAD^+^ and how this might be amplified during intestinal inflammation needs to be further investigated.

In summary, current data suggest that P2X7-mediated cell death constitutes a crucial factor for the homeostatic regulation of several T cell subsets that play essential roles in the regulation of immune responses. P2X7 can therefore be viewed as an important regulator of T cell functions, notably through the induction of P2X7-dependent T cell death. Still, dependent on the level of expression of P2X7 and on the amount of available extracellular ATP and/or NAD^+^, P2X7 may also conceivably regulate T cell activation and/or function without necessarily inducing cell death. In the following section, we will highlight the role of P2X7 as a co-stimulatory partner during the activation of T cells.

## P2X7-Mediated Autocrine Stimulation of Human and Mouse T Cells

It has become clear that ATP can be released from living cells in different physiological and pathophysiological situations (68). For instance, hypertonic and mechanical stress induces deformation of the plasma membrane that can lead to the liberation of ATP into the extracellular milieu from many cell types. When applied to Jurkat T cells, hypertonic stress leads to the rapid release of extracellular ATP, to the augmentation of intracellular Ca^2+^, to mitogen-activated protein kinase (MAPK) activation, and to enhanced expression of interleukin 2 (IL-2) (69). Studies addressing the underlying mechanisms of ATP release identified pannexin 1 hemichannels as a critical mediator of ATP release in response to hypertonic stress (70). Interestingly, TCR stimulation by itself triggered the release of ATP in a pannexin-1 dependent manner (71, 72). ATP released during TCR stimulation activates P2X7, which sustains MAPK signaling, stimulating IL-2 expression and enhancing T cell proliferation. Further, antagonism of P2X7 during TCR activation blunted MAPK signaling and induced a transcriptional program characteristic for T cell anergy (71). The importance of this regulatory mechanism has been evaluated in a mouse model of diabetes that is based on the adoptive transfer of hemagglutinin (HA)-specific TCR-transgenic T cells into Rag2^-/-^ mice expressing HA under control of the insulin promoter (73). Simultaneous injection of oxidized ATP (oATP) to antagonize of P2X7 *in vivo* prevented the onset of diabetes (71). As oATP is not an entirely specific antagonist for P2X7, the implication of other P2X receptors in this observation cannot be completely ruled out. Indeed, other ATP-sensitive P2X receptors can also contribute to purinergic T cell stimulation. For instance, P2X1 and P2X4 were shown to translocate into the immunological synapse, together with pannexin 1, following TCR stimulation. In this context, small molecule antagonists of P2X1 and P2X4 had similar inhibitory effects on T cell activation as P2X7 antagonists (74). However, the specificity of some of these small inhibitory molecules is uncertain, precluding any definitive interpretation of the specific role of each P2X receptor on T cell stimulation. The generation of highly specific biological antagonistic nanobodies will certainly help to gain new insights on their specific roles (75).

## Discussion on the Function of P2X7 on Human and Murine T Cells

**Figure 3:**
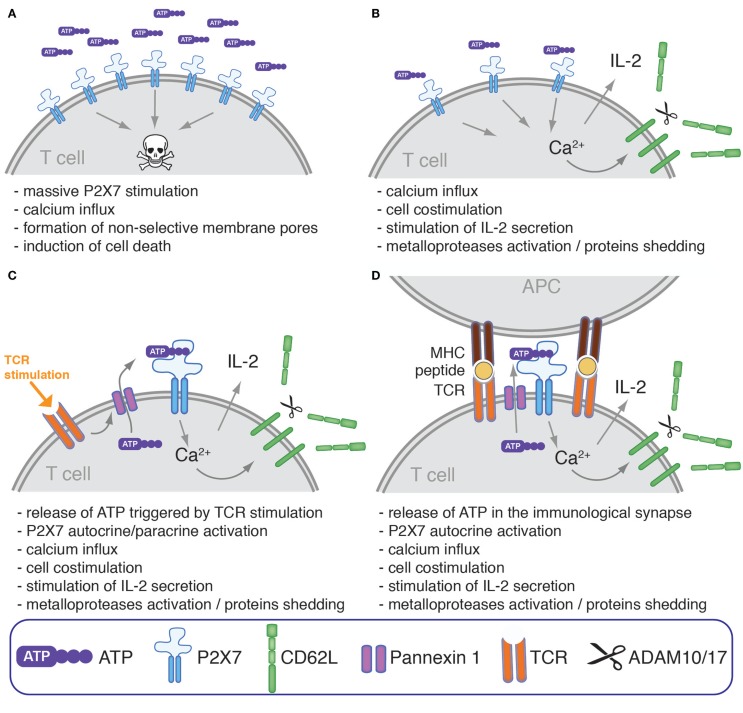
**Modulation of human T cells phenotype, response to mitogenic stimulation, and survival by ATP and P2X7**. P2X7-stimulation on the surface of human T cell may have different consequences which could hypothetically depend on the extracellular concentration of ATP, P2X7 density, expression of CD39 and other ATPases, on the nature of T cell subsets, and on their activation status. **(A)** High concentration of extracellular ATP have been reported to culminate, at least *in vitro*, in the induction of cell death possibly through the induction of massive membrane depolarization and permeabilization. **(B)** At lower ATP concentration and/or if cells express a low level of surface P2X7 and/or a high level of ATP-catabolizing enzymes, P2X7-activation could participate instead in T cell costimulation by enhancing the intracellular level of Ca^2+^, an universal second messenger (76, 78). This may result in the stimulation of NFAT, MAPKs, and IL-2 secretion (69, 70, 72), and in the activation of metalloproteases that catalyze the shedding of CD62L and of other cell surface proteins (79). **(C)** In some studies, TCR stimulation by mitogenic activators was shown to promote ATP release through pannexin 1 favoring autocrine/paracrine T cell co-stimulation (69, 70, 72). **(D)** TCR stimulation was also found to stimulate the translocation of pannexin 1 at the immunological synapse together with P2X1 and P2X4 (80). P2X7 is also found within the immunological synapse even if it is not actively translocated within the immunological synapse and remains instead uniformly distributed across the cell surface. This mechanism may thus serve to provide a tonic co-stimulatory signal during physiological activation of a T cell in contact with antigen-presenting cells by promoting the local release of ATP within the immunological synapse, and the local activation of P2X receptors expressed by T cell as well as by antigen-presenting cells.

Much work in mouse models has led to the conclusion that P2X7 regulates many aspects of mouse T cell biology. This multifaceted ATP-gated ion channel is expressed at high levels on different T cell subsets that are important for the regulation of immune responses such as Tregs, NKT cells, or Tfh cells. The question that may be raised is how P2X7 delivers signals that regulate such distinct events as T cell activation, proliferation, phenotype, cytokine production, and cell death (Figure 1). A possible answer might reside in the temporal and spatial regulation of the availability of extracellular purinergic ligands and on the density of P2X7 expression at the cell surface. Long-lasting whole-cell activation of P2X7 by high concentrations of ATP or NAD^+^ in cells expressing high levels of P2X7 might promote cell death, whereas short pulses and/or low global amounts of ATP or its restricted presence at only one side of a cell, such as would occur in the immunological synapse, might instead promote TCR-mediated activation of T cells (Figure 3). Hints in this direction have been provided by studies showing that low concentrations of ATP co-stimulate proliferation of human peripheral blood T lymphocytes (76), Jurkat cell (74) and human activated/memory CD4^+^ T cells (77), whereas 1 mM ATP induces T cell death (76, 77). It may be stressed here that ATP-triggered cell death of human T cells differs greatly in magnitude and kinetics as compared to mouse T cells. ATP-triggered cell death of human T cell is observed in smaller fractions of cells and only when exposed to millimolar ATP concentrations for a few days. For comparison, 1–30 μM NAD^+^ or 100–300 μM of ATP suffice to induce cell death of the vast majority of mouse T cells within an hour. These differences may be linked to the lower expression level of P2X7 at the surface of human T cells and to the absence of a highly potent T cell-specific P2X7 variant in humans analogous to the mouse P2X7k variant. Hence, further studies will be required to clarify the physiological roles of P2X7 in the regulation of human T cell death, homeostasis, and activation.

## Conflict of Interest Statement

Friedrich Koch-Nolte and Friedrich Haag receive royalties from sales of antibodies developed in the lab via MediGate GmbH, a 100% subsidiary of the University Medical Center, Hamburg. The other co-authors declare that the research was conducted in the absence of any commercial or financial relationships that could be construed as a potential conflict of interest.

## References

[1] Bartlett R Stokes L Sluyter R. The P2X7 receptor channel: recent developments and the use of P2X7 antagonists in models of disease. Pharmacol Rev (2014) 66:638–75. 10.1124/pr.113.008003 24928329

[2] Seman M Adriouch S Scheuplein F Krebs C Freese D Glowacki G NAD-induced T cell death: ADP-ribosylation of cell surface proteins by ART2 activates the cytolytic P2X7 purinoceptor. Immunity (2003) 19:571–82. 10.1016/S1074-7613(03)00266-8 14563321

[3] Adriouch S Bannas P Schwarz N Fliegert R Guse AH Seman M ADP-ribosylation at R125 gates the P2X7 ion channel by presenting a covalent ligand to its nucleotide binding site. FASEB J (2007) 22:861–9. 10.1096/fj.07-9294com 17928361

[4] Scheuplein F Schwarz N Adriouch S Krebs C Bannas P Rissiek B NAD+ and ATP released from injured cells induce P2X7-dependent shedding of CD62L and externalization of phosphatidylserine by murine T cells. J Immunol (2009) 182:2898–908. 10.4049/jimmunol.0801711 19234185

[5] Hubert S Rissiek B Klages K Huehn J Sparwasser T Haag F Extracellular NAD+ shapes the Foxp3+ regulatory T cell compartment through the ART2-P2X7 pathway. J Exp Med (2010) 207:2561–8. 10.1084/jem.20091154 20975043 PMC2989765

[6] Rissiek B Danquah W Haag F Koch-Nolte F. Technical advance: a new cell preparation strategy that greatly improves the yield of vital and functional Tregs and NKT cells. J Leukoc Biol (2014) 95:543–9. 10.1189/jlb.0713407 24212099

[7] Haag F Koch-Nolte F Kühl M Lorenzen S Thiele HG. Premature stop codons inactivate the RT6 genes of the human and chimpanzee species. J Mol Biol (1994) 243:537–46. 10.1006/jmbi.1994.1680 7966280

[8] Schwarz N Fliegert R Adriouch S Seman M Guse AH Haag F Activation of the P2X7 ion channel by soluble and covalently bound ligands. Purinergic Signal (2009) 5:139–49. 10.1007/s11302-009-9135-5 19255877 PMC2686825

[9] Lévesque SA Kukulski F Enjyoji K Robson SC Sévigny J. NTPDase1 governs P2X7-dependent functions in murine macrophages. Eur J Immunol (2010) 40:1473–85. 10.1002/eji.200939741 20201036 PMC3045779

[10] Kuhny M Hochdörfer T Ayata CK Idzko M Huber M. CD39 is a negative regulator of P2X7-mediated inflammatory cell death in mast cells. Cell Commun Signal (2014) 12:40. 10.1186/s12964-014-0040-3 25184735 PMC4110707

[11] Krebs C Adriouch S Braasch F Koestner W Leiter EH Seman M CD38 controls ADP-ribosyltransferase-2-catalyzed ADP-ribosylation of T cell surface proteins. J Immunol (2005) 174:3298–305. 10.4049/jimmunol.174.6.3298 15749861

[12] Deaglio S Dwyer KM Gao W Friedman D Usheva A Erat A Adenosine generation catalyzed by CD39 and CD73 expressed on regulatory T cells mediates immune suppression. J Exp Med (2007) 204:1257–65. 10.1084/jem.20062512 17502665 PMC2118603

[13] Magnone M Bauer I Poggi A Mannino E Sturla L Brini M NAD+ levels control Ca2+ store replenishment and mitogen-induced increase of cytosolic Ca2+ by Cyclic ADP-ribose-dependent TRPM2 channel gating in human T lymphocytes. J Biol Chem (2012) 287:21067–81. 10.1074/jbc.M111.324269 22547068 PMC3375530

[14] Di Virgilio F Boeynaems J-M Robson SC. Extracellular nucleotides as negative modulators of immunity. Curr Opin Pharmacol (2009) 9:507–13. 10.1016/j.coph.2009.06.021 19628431 PMC4158611

[15] Haag F Adriouch S Braß A Jung C Möller S Scheuplein F Extracellular NAD and ATP: partners in immune cell modulation. Purinergic Signal (2007) 3:71–81. 10.1007/s11302-006-9038-7 18404420 PMC2096762

[16] Aymeric L Apetoh L Ghiringhelli F Tesniere A Martins I Kroemer G Tumor cell death and ATP release prime dendritic cells and efficient anticancer immunity. Cancer Res (2010) 70:855–8. 10.1158/0008-5472.CAN-09-3566 20086177

[17] Garg AD Krysko DV Verfaillie T Kaczmarek A Ferreira GB Marysael T A novel pathway combining calreticulin exposure and ATP secretion in immunogenic cancer cell death. EMBO J (2012) 31:1062–79. 10.1038/emboj.2011.497 22252128 PMC3298003

[18] Adriouch S Hubert S Pechberty S Koch-Nolte F Haag F Seman M. NAD+ released during inflammation participates in T cell homeostasis by inducing ART2-mediated death of naive T cells in vivo. J Immunol (2007) 179:186–94. 10.4049/jimmunol.179.1.186 17579037

[19] Taylor SRJ Gonzalez-Begne M Dewhurst S Chimini G Higgins CF Melvin JE Sequential shrinkage and swelling underlie P2X7-stimulated lymphocyte phosphatidylserine exposure and death. J Immunol (2008) 180:300–8. 10.4049/jimmunol.180.1.300 18097031

[20] Adriouch S Ohlrogge W Haag F Koch-Nolte F Seman M. Rapid induction of naive T cell apoptosis by ecto-nicotinamide adenine dinucleotide: requirement for mono(ADP-ribosyl)transferase 2 and a downstream effector. J Immunol (2001) 167:196–203. 10.4049/jimmunol.167.1.196 11418649

[21] Courageot M-P Lépine S Hours M Giraud F Sulpice J-C. Involvement of sodium in early phosphatidylserine exposure and phospholipid scrambling induced by P2X7 purinoceptor activation in thymocytes. J Biol Chem (2004) 279:21815–23. 10.1074/jbc.M401426200 14996828

[22] Bratton DL Fadok VA Richter DA Kailey JM Guthrie LA Henson PM. Appearance of phosphatidylserine on apoptotic cells requires calcium-mediated nonspecific flip-flop and is enhanced by loss of the aminophospholipid translocase. J Biol Chem (1997) 272:26159–65. 10.1074/jbc.272.42.26159 9334182

[23] Bevers EM Williamson PL. Phospholipid scramblase: an update. FEBS Lett (2010) 584:2724–30. 10.1016/j.febslet.2010.03.020 20302864

[24] Mackenzie AB Young MT Adinolfi E Surprenant A Pseudoapoptosis induced by brief activation of ATP-gated P2X7 receptors. J Biol Chem (2005) 280:33968–76 10.1074/jbc.M502705200 15994333

[25] Becker D Woltersdorf R Boldt W Schmitz S Braam U Schmalzing G The P2X7 carboxyl tail is a regulatory module of P2X7 receptor channel activity. J Biol Chem (2008) 283:25725–34. 10.1074/jbc.M803855200 18617511

[26] Shoji KF Sáez PJ Harcha PA Aguila HL Sáez JC. Pannexin1 channels act downstream of P2X 7 receptors in ATP-induced murine T-cell death. Channels (Austin) (2014) 8:142–56. 10.4161/chan.28122 24590064 PMC4048303

[27] Ousingsawat J Wanitchakool P Kmit A Romao AM Jantarajit W Schreiber R Anoctamin 6 mediates effects essential for innate immunity downstream of P2X7 receptors in macrophages. Nat Commun (2015) 6:6245. 10.1038/ncomms7245 25651887

[28] Gu B Bendall LJ Wiley JS. Adenosine triphosphate-induced shedding of CD23 and L-selectin (CD62L) from lymphocytes is mediated by the same receptor but different metalloproteases. Blood (1998) 92:946–51. 9680363

[29] Moon H Na H-Y Chong KH Kim TJ. P2X7 receptor-dependent ATP-induced shedding of CD27 in mouse lymphocytes. Immunol Lett (2006) 102:98–105. 10.1016/j.imlet.2005.08.004 16207496

[30] Garbers C Janner N Chalaris A Moss ML Floss DM Meyer D Species specificity of ADAM10 and ADAM17 proteins in interleukin-6 (IL-6) trans-signaling and novel role of ADAM10 in inducible IL-6 receptor shedding. J Biol Chem (2011) 286:14804–11. 10.1074/jbc.M111.229393 21454673 PMC3083187

[31] Le Stunff H Auger R Kanellopoulos J Raymond M-N. The Pro-451 to Leu polymorphism within the C-terminal tail of P2X7 receptor impairs cell death but not phospholipase D activation in murine thymocytes. J Biol Chem (2004) 279:16918–26. 10.1074/jbc.M313064200 14761980

[32] Young MT Pelegrín P Surprenant A. Identification of Thr283 as a key determinant of P2X7 receptor function. Br J Pharmacol (2006) 149:261–8. 10.1038/sj.bjp.0706880 16940988 PMC2014266

[33] Sorge RE Trang T Dorfman R Smith SB Beggs S Ritchie J Genetically determined P2X7 receptor pore formation regulates variability in chronic pain sensitivity. Nat Med (2012) 18:595–9. 10.1038/nm.2710 22447075 PMC3350463

[34] Syberg S Petersen S Beck Jensen J-E Gartland A Teilmann J Chessell I Genetic background strongly influences the bone phenotype of P2X7 receptor knockout mice. J Osteoporos (2012) 2012:391097. 10.1155/2012/391097 22934234 PMC3425798

[35] Sluyter R Stokes L. Significance of P2X7 receptor variants to human health and disease. Recent Pat DNA Gene Seq (2011) 5:41–54. 10.2174/187221511794839219 21303345

[36] Adriouch S Dox C Welge V Seman M Koch-Nolte F Haag F. Cutting edge: a natural P451L mutation in the cytoplasmic domain impairs the function of the mouse P2X7 receptor. J Immunol (2002) 169:4108–12. 10.4049/jimmunol.169.8.4108 12370338

[37] Nicke A Kuan Y-H Masin M Rettinger J Marquez-Klaka B Bender O A functional P2X7 splice variant with an alternative transmembrane domain 1 escapes gene inactivation in P2X7 knock-out mice. J Biol Chem (2009) 284:25813–22. 10.1074/jbc.M109.033134 19546214 PMC2757983

[38] Schwarz N Drouot L Nicke A Fliegert R Boyer O Guse AH Alternative splicing of the N-terminal cytosolic and transmembrane domains of P2X7 controls gating of the ion channel by ADP-ribosylation. PLoS One (2012) 7:e41269. 10.1371/journal.pone.0041269 22848454 PMC3407210

[39] Xu XJ Boumechache M Robinson LE Marschall V Górecki DC Masin M Splice-variants of the P2X7 receptor reveal differential agonist-dependence and functional coupling with pannexin-1. J Cell Sci (2012) 125(Pt 16):3776–89. 10.1242/jcs.099374 22553206

[40] Hong S Schwarz N Braß A Seman M Haag F Koch-Nolte F Differential regulation of P2X7 receptor activation by extracellular nicotinamide adenine dinucleotide and ecto-ADP-ribosyltransferases in murine macrophages and T cells. J Immunol (2009) 183:578–92. 10.4049/jimmunol.0900120 19542469 PMC2768492

[41] Solle M Labasi J Perregaux DG Stam E Petrushova N Koller BH Altered cytokine production in mice lacking P2X(7) receptors. J Biol Chem (2001) 276:125–32. 10.1074/jbc.M006781200 11016935

[42] Ke HZ Qi H Weidema AF Zhang Q Panupinthu N Crawford DT Deletion of the P2X7 nucleotide receptor reveals its regulatory roles in bone formation and resorption. Mol Endocrinol (2003) 17:1356–67. 10.1210/me.2003-0021 12677010

[43] Chessell IP Hatcher JP Bountra C Michel AD Hughes JP Green P Disruption of the P2X7 purinoceptor gene abolishes chronic inflammatory and neuropathic pain. Pain (2005) 114:386–96. 10.1016/j.pain.2005.01.002 15777864

[44] Sim JA Young MT Sung H-Y North RA Surprenant A. Reanalysis of P2X7 receptor expression in rodent brain. J Neurosci (2004) 24:6307–14. 10.1523/JNEUROSCI.1469-04.2004 15254086 PMC6729549

[45] Taylor SRJ Gonzalez-Begne M Sojka DK Richardson JC Sheardown SA Harrison SM Lymphocytes from P2X7-deficient mice exhibit enhanced P2X7 responses. J Leukoc Biol (2009) 85:978–86. 10.1189/jlb.0408251 19276178 PMC2698584

[46] Sharp AJ Polak PE Simonini V Lin SX Richardson JC Bongarzone ER P2x7 deficiency suppresses development of experimental autoimmune encephalomyelitis. J Neuroinflammation (2008) 5:33. 10.1186/1742-2094-5-33 18691411 PMC2518548

[47] Chen L Brosnan CF Exacerbation of experimental autoimmune encephalomyelitis in P2X7R-/- mice: evidence for loss of apoptotic activity in lymphocytes. J Immun (2006) 176:3115–26 10.4049/jimmunol.176.5.3115 16493071

[48] Freedman BD Liu QH Gaulton G Kotlikoff MI Hescheler J Fleischmann BK. ATP-evoked Ca2+ transients and currents in murine thymocytes: possible role for P2X receptors in death by neglect. Eur J Immunol (1999) 29:1635–46. 10.1002/(SICI)1521-4141(199905)29:05<1635::AID-IMMU1635>3.3.CO;2-2 10359118

[49] Frascoli M Marcandalli J Schenk U Grassi F. Purinergic P2X7 receptor drives T cell lineage choice and shapes peripheral γδ cells. J Immunol (2012) 189:174–80. 10.4049/jimmunol.1101582 22649196

[50] Haag F Freese D Scheublein F Ohlrogge W Adriouch S Seman M T cells of different developmental stages differ in sensitivity to apoptosis induced by extracellular NAD. Dev Immunol (2002) 9:197–202. 10.1080/10446670310001593514 15144016 PMC2276114

[51] Kahl S Nissen M Girisch R Duffy T Leiter EH Haag F Metalloprotease-mediated shedding of enzymatically active mouse ecto-ADP-ribosyltransferase ART2.2 upon T cell activation. J Immunol (2000) 165:4463–9. 10.4049/jimmunol.165.8.4463 11035085

[52] Aswad F Dennert G. P2X7 receptor expression levels determine lethal effects of a purine based danger signal in T lymphocytes. Cell Immunol (2006) 243:58–65. 10.1016/j.cellimm.2006.12.003 17286969 PMC1913480

[53] Rissiek B Haag F Boyer O Koch-Nolte F Adriouch S. ADP-ribosylation of P2X7: a matter of life and death for regulatory T cells and natural killer T cells. Curr Top Microbiol Immunol (2014) 384:107–26. 10.1007/82_2014_420 25048544

[54] Aswad F Kawamura H Dennert G. High sensitivity of CD4+CD25+ regulatory T cells to extracellular metabolites nicotinamide adenine dinucleotide and ATP: a role for P2X7 receptors. J Immunol (2005) 175:3075–83. 10.4049/jimmunol.175.5.3075 16116196

[55] Kawamura H Aswad F Minagawa M Govindarajan S Dennert G. P2X7 receptors regulate NKT cells in autoimmune hepatitis. J Immunol (2006) 176:2152–60. 10.4049/jimmunol.176.4.2152 16455971

[56] Koch-Nolte F Reyelt J Schössow B Schwarz N Scheuplein F Rothenburg S Single domain antibodies from llama effectively and specifically block T cell ecto-ADP-ribosyltransferase ART2.2 in vivo. FASEB J (2007) 21:3490–8. 10.1096/fj.07-8661com 17575259

[57] Bao L Locovei S Dahl G. Pannexin membrane channels are mechanosensitive conduits for ATP. FEBS Lett (2004) 572:65–8. 10.1016/j.febslet.2004.07.009 15304325

[58] Bruzzone S Guida L Zocchi E Franco L De Flora A. Connexin 43 hemi channels mediate Ca2+-regulated transmembrane NAD+ fluxes in intact cells. FASEB J (2001) 15:10–2. 10.1096/fj.00-0566fje 11099492

[59] Scheuplein F Rissiek B Driver JP Chen Y-G Koch-Nolte F Serreze DV. A recombinant heavy chain antibody approach blocks ART2 mediated deletion of an iNKT cell population that upon activation inhibits autoimmune diabetes. J Autoimmun (2010) 34:145–54. 10.1016/j.jaut.2009.08.012 19796917 PMC2822066

[60] Takeda K Hayakawa Y Van Kaer L Matsuda H Yagita H Okumura K. Critical contribution of liver natural killer T cells to a murine model of hepatitis. Proc Natl Acad Sci U S A (2000) 97:5498–503. 10.1073/pnas.040566697 10792025 PMC25857

[61] Hofman P Cherfils-Vicini J Bazin M Ilie M Juhel T Hebuterne X Genetic and pharmacological inactivation of the purinergic P2RX7 receptor dampens inflammation but increases tumor incidence in a mouse model of colitis-associated cancer. Cancer Res (2015) 75:835–45. 10.1158/0008-5472.CAN-14-1778 25564520

[62] Ghiringhelli F Apetoh L Tesniere A Aymeric L Ma Y Ortiz C Activation of the NLRP3 inflammasome in dendritic cells induces IL-1beta-dependent adaptive immunity against tumors. Nat Med (2009) 15:1170–8. 10.1038/nm.2028 19767732

[63] Adinolfi E Capece M Franceschini A Falzoni S Giuliani AL Rotondo A Accelerated tumor progression in mice lacking the ATP receptor P2X7. Cancer Res (2015) 75:635–44. 10.1158/0008-5472.CAN-14-1259 25542861

[64] Heiss K Jänner N Mähnss B Schumacher V Koch-Nolte F Haag F High sensitivity of intestinal CD8+ T cells to nucleotides indicates P2X7 as a regulator for intestinal T cell responses. J Immunol (2008) 181:3861–9. 10.4049/jimmunol.181.6.3861 18768840

[65] Iwata M Hirakiyama A Eshima Y Kagechika H Kato C Song S-Y. Retinoic acid imprints gut-homing specificity on T cells. Immunity (2004) 21:527–38. 10.1016/j.immuni.2004.08.011 15485630

[66] Coombes JL Siddiqui KRR Arancibia-Cárcamo CV Hall J Sun C-M Belkaid Y A functionally specialized population of mucosal CD103+ DCs induces Foxp3+ regulatory T cells via a TGF-beta and retinoic acid-dependent mechanism. J Exp Med (2007) 204:1757–64. 10.1084/jem.20070590 17620361 PMC2118683

[67] Proietti M Cornacchione V Rezzonico Jost T Romagnani A Faliti CE Perruzza L ATP-gated ionotropic P2X7 receptor controls follicular T helper cell numbers in Peyer’s patches to promote host-microbiota mutualism. Immunity (2014) 41:789–801. 10.1016/j.immuni.2014.10.010 25464855

[68] Praetorius HA Leipziger J ATP release from non-excitable cells. Purinergic Signal (2009) 5:433–46 10.1007/s11302-009-9146-2 19301146 PMC2776134

[69] Loomis WH Namiki S Ostrom RS Insel PA Junger WG. Hypertonic stress increases T cell interleukin-2 expression through a mechanism that involves ATP release, P2 receptor, and p38 MAPK activation. J Biol Chem (2003) 278:4590–6. 10.1074/jbc.M207868200 12464620

[70] Woehrle T Yip L Manohar M Sumi Y Yao Y Chen Y Hypertonic stress regulates T cell function via pannexin-1 hemichannels and P2X receptors. J Leukoc Biol (2010) 88:1181–9. 10.1189/jlb.0410211 20884646 PMC2996895

[71] Schenk U Westendorf AM Radaelli E Casati A Ferro M Fumagalli M Purinergic control of T cell activation by ATP released through pannexin-1 hemichannels. Sci Signal (2008) 1:ra6–6. 10.1126/scisignal.1160583 18827222

[72] Yip L Woehrle T Corriden R Hirsh M Chen Y Inoue Y Autocrine regulation of T-cell activation by ATP release and P2X7 receptors. FASEB J (2009) 23:1685–93. 10.1096/fj.08-126458 19211924 PMC2718802

[73] Sarukhan A Garcia C Lanoue A Boehmer von H. Allelic inclusion of T cell receptor alpha genes poses an autoimmune hazard due to low-level expression of autospecific receptors. Immunity (1998) 8:563–70. 10.1016/S1074-7613(00)80561-0 9620677

[74] Woehrle T Yip L Elkhal A Sumi Y Chen Y Yao Y Pannexin-1 hemichannel-mediated ATP release together with P2X1 and P2X4 receptors regulate T-cell activation at the immune synapse. Blood (2010) 116:3475–84. 10.1182/blood-2010-04-277707 20660288 PMC2981474

[75] Rissiek B Koch-Nolte F Magnus T. Nanobodies as modulators of inflammation: potential applications for acute brain injury. Front Cell Neurosci (2014) 8:344. 10.3389/fncel.2014.00344 25374510 PMC4204521

[76] Adinolfi E Pizzirani C Idzko M Panther E Norgauer J Virgilio F P2X7 receptor: death or life? Purinergic Signal (2005) 1:219–27. 10.1007/s11302-005-6322-x 18404507 PMC2096546

[77] Trabanelli S Očadlíková D Gulinelli S Curti A Salvestrini V Vieira RP Extracellular ATP exerts opposite effects on activated and regulatory CD4+ T cells via purinergic P2 receptor activation. J Immunol (2012) 189:1303–10. 10.4049/jimmunol.1103800 22753942

[78] Baricordi OR Ferrari D Melchiorri L Chiozzi P Hanau S Chiari E An ATP-activated channel is involved in mitogenic stimulation of human T lymphocytes. Blood (1996) 87:682–90. 8555491

[79] Burnstock G Boeynaems J-M Purinergic signalling and immune cells. Purinergic Signal (2014) 10:529–64 10.1007/s11302-014-9427-2 25352330 PMC4272370

[80] Junger WG. Immune cell regulation by autocrine purinergic signalling. Nat Rev Immunol (2011) 11:201–12. 10.1038/nri2938 21331080 PMC4209705

